# Clinical value of MRI, serum SCCA, and CA125 levels in the diagnosis of lymph node metastasis and para-uterine infiltration in cervical cancer

**DOI:** 10.1186/s12957-021-02448-3

**Published:** 2021-12-09

**Authors:** Chao Ran, Jian Sun, Yunhui Qu, Na Long

**Affiliations:** Department of Medical Imaging, Affiliated Yantai Yuhuangding Hospital of Qingdao University, No.20 Yuhuangding East Road, Zhifu District, Yantai, 264000 China

**Keywords:** MRI, Tumor markers, Cervical cancer, Preoperative diagnosis

## Abstract

**Background:**

Cervical cancer shows great differences in depth of invasion, metastasis, and other biological behaviors. The location of the lesion is special, so it is usually difficult to determine the clinical stage. This study aimed to explore the clinical value of magnetic resonance imaging (MRI) and tumor serum markers for the preoperative diagnosis of cervical cancer lymph node metastasis and para-uterine invasion.

**Methods:**

A total of 200 patients with cervical cancer admitted to our hospital from January 2019 to January 2020 were collected as the research subjects. Comparing the diagnosis results of preoperative MRI scan, serum tumor markers, and postoperative pathological examination using single factor comparison, we determined the MRI scan results, the comprehensive matching rate between serum tumor markers (squamous cell carcinoma antigen (SCCA), carbohydrate antigen 125 (CA125)) and postoperative pathological results, and the differences of sensitivity, specificity, and accuracy in the prediction of lymph node metastasis and para-uterine infiltration of cervical cancer.

**Results:**

The levels of SCCA and CA125 in patients with para-uterine invasion and lymph node metastasis were higher than those of patients without invasion and metastasis. Among them, the level of SCCA was significantly different (*P*<0.05). The level of CA125 was not statistically significant (*P*>0.05), so MRI combined with serum SCCA was selected for combined diagnosis in the later period. The sensitivity, specificity, and accuracy of MRI diagnosis of cervical cancer and para-uterine infiltrating lymph node metastasis and metastasis were 55.2, 91.6, and 89.5% and 55.2, 91.6, and 89.5%, respectively. These data in MRI combined with serum SCCA were 76.3, 95.3, and 94.3% and 63.2, 96.0, and 95.1%, respectively. The accuracy of tumor markers combined with MRI in the diagnosis of cervical cancer lymph node metastasis and para-uterine invasion was higher than that of MRI.

**Conclusions:**

MRI combined with serum SCCA can more accurately identify cervical cancer lymph node metastasis and para-uterine invasion compared with MRI alone. Tumor marker combined with MRI diagnosis is an important auxiliary method for cervical cancer treatment and can provide comprehensive and reliable clinical evidence for evaluation before cervical cancer surgery.

## Background

In the gynecological clinical practice, cervical cancer is the second most common cancer worldwide [1], second only to breast cancer [2], ranking second among cancer-related causes of death in women in developing countries [3]. There are about 510,000 new cases of cervical cancer every year, accounting for 9.8% of all cancers. There are about 100,000 new cases every year in China. Patients with cervical cancer tend to be younger [4], among which the number of those with precancerous lesions under 35 nearly halves the total. Therefore, the demand on the diagnosis and treatment for cervical cancer is very urgent.

Lymph node metastasis (LNM), one of the major modes of metastasis in cervical cancer, is also one of the important factors in determining the prognosis and treatment plan. Federation Internationale of Gynecologie and Obstetrigue (FIGO) 2018 [5] listed LNM in cervical cancer as an independent factor affecting the prognosis in patients [6], with 17~33% LNM rate in early cervical cancer [7]. The 5-year survival in patients with early cervical cancer without LNM is up to 90%, while 5-year survival in patients with LNM deteriorated rapidly, at only 65% (*P* <0.05) [8]. Due to LNM in some patients with early cervical cancer [9], patients might have been over-treated with more adverse reactions and more complications [10]; therefore, it is crucial to discover the treatment strategies and the prediction of prognosis for accurate prediction of LNM in patients with cervical cancer.

Para-uterine infiltration includes para-uterine soft tissue involvement, para-uterine LNM, and tumor emboli in para-uterine vessels [11]. It is an important prognostic factor in cervical cancer, which is associated with a high risk of LNM [12]. Therefore, it is important to assess cervical involvement before developing a treatment plan.

Currently, magnetic resonance imaging (MRI) is often used to predict pelvic LNM clinically [13]. MRI is a non-invasive scanning technique with the advantages of distinguishing inflammatory hyperplasia and finding LNM, which is characterized by multi-sequence, multi-parameter, and multi-directional in imaging [14]. MRI not only clearly identifies para-uterine infiltration and LNM in cervical cancer, but also has high resolution to soft tissues of the uterus and cervix. It is able to accurately determine tumor volume, para-uterine infiltration, and LNM because of no ionizing radiation [15, 16], which, thus, can be taken as one of the most respected imaging technologies in cervical cancer [17, 18]. Although these imaging methods have potential in predicting LNM, they are not ideal due to morphological indexes such as size and shape with low sensitivity (38–56%) [19, 20], which may be due to micrometastatic lymph nodes. Therefore, a significant proportion of patients with lymph node metastasis due to cervical cancer are not identified.

SCCA was first discovered in the cytoplasm of liver metastases in patients with cervical cancer. It has specific protease inhibitory properties and can be controlled by participating in cell apoptosis. SCCA in peripheral blood can hardly be detected under normal physiological conditions, and the serum level of SCCA in patients with benign tumors and adenocarcinomas is also low, so it has been confirmed as a specific tumor marker for squamous cell carcinoma [21]. CA125 is a typical high-molecular glycoprotein, usually distributed in the epithelial cells of the body cavity. It has been confirmed that the expression of CA125 in neoplastic diseases is higher than that of non-neoplastic diseases [22, 23].

Serum SCCA and CA125 are clinically recognized as the preferred markers for screening cervical squamous carcinoma, which can assist in the diagnosis and early detection of cervical cancer. Serum tumor markers are associated with the increase of in vivo squamous cell cancer tissues, which can initially predict the situation of LNM in cervical cancer [14], with advantages of simple operation and good compliance. It can be used as an extensive tool in the diagnosis and treatment of LNM and in the prognostic evaluation of malignant tumors in recent years [24]. However, due to the low specificity of para-uterine infiltration and LNM with serum tumor markers, imaging examination is still needed. Studies have pointed out that a variety of markers are highly sensitive in jointly diagnosing cervical cancer [25].

Therefore, it is necessary to explore valuable examination methods for more accurate clinical stages, to understand LNM and peri-uterine infiltration in detail, and find out the correct clinical intervention. This study intends to explore the clinical value and accuracy of MRI, tumor markers (serum SCCA and CA125), and their combination in the diagnosis of LNM and para-uterine infiltration in cervical cancer.

## Materials and methods

### Research objects

This single-center retrospective study was conducted in accordance with the principles of the Declaration of Helsinki. The study protocol was approved by the ethics committee of our hospital. Since this study is a retrospective analysis, the patient’s consent for inclusion was abandoned.

Inclusion criteria include (A) patients who underwent total hysterectomy combined with pelvic lymphatic dissection and were confirmed to be cervical squamous cell carcinoma by pathological examination of the specimen; (B) patients with FIGO stage ≥ IB stage (in stages IB1 and IB2, the clinically visible lesions are confined to the cervix, or the preclinical lesions are larger than that of stage IA); (C) patients who received pelvic MRI plain scan + enhanced examination 1 to 2 weeks before treatment; (D) patients with single or multiple tests for tumor markers (serum SCCA and CA125) before treatment; (E) patients with surgical treatment as the initial treatment; and (F) patients without any preoperative chemoradiotherapy. Exclusion criteria include (A) patients who received preoperative treatment (neoadjuvant chemotherapy, radiotherapy, or coning); (B) patients lacking any MRI sequence, including T2WI, DWI, and CT1W MRI without fat suppression; (C) patients with poor MRI image quality caused by motion artifacts; (D) patients with lesions that are not visible with the above MRI sequences; and (E) patients with other diseases that could lead to high serum tumor markers.

A total of 200 patients with cervical cancer who received treatment in our hospital from January 2019 to January 2021 were collected retrospectively as the observation group, which were diagnosed by pathological histopathology, including 152 patients with stage I, 39 patients with stage II, and 9 patients with stage III.

**Fig. 1:**
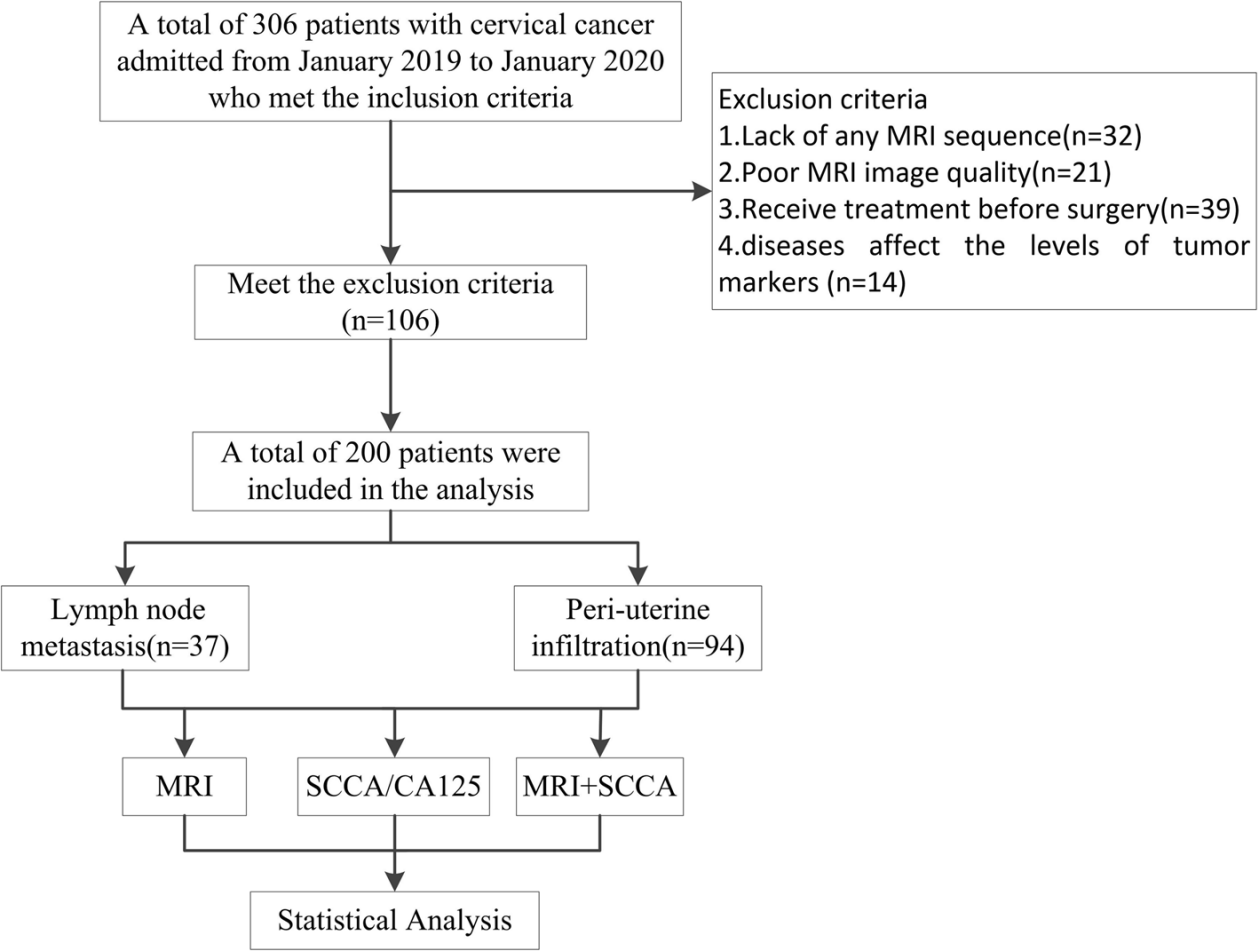
Flowchart of patient selection

All patients have acquired preoperative characteristics, including age, LN signal intensity (uniform or non-uniform T2WI), LN boundaries (smooth or burr), LN size, and tumor size. LN signal intensity and boundaries were evaluated by a radiologist (Chao Ran) with 7 years of experience in pelvic MRI diagnosis and a senior radiologist (Na Long) with 20 years of experience in pelvic MRI diagnosis. See Fig. 1.

### Obvervational indexes

Patients’ age, clinical stage, preoperative SCCA level, CA125 level, MRI diagnosis, surgical approach, postoperative pathological type, and LNM were recorded. The sensitivity, specificity, accuracy, positive prediction values, and negative prediction values of pelvic LNM diagnosis using MRI alone were calculated according to the postoperative pathological results as the gold criteria for diagnosing LNM. The receiver operating characteristic (ROC) curve of LNM was drawn with SCCA and CA125 test values, and the area under the curve, the optimal critical value, Youden’s index, sensitivity, and specificity of SCCA and CA125 in the diagnosis of pelvic LNM were evaluated. The optimal critical values of SCCA and CA125 in the diagnosis of pelvic LNM were combined to diagnose MRI, to calculate the sensitivity, specificity, accuracy, and positive predictive value and negative predictive value.

### MRI examination

MRI was performed about one week before surgery. Prior to examination, all the patients were required to drink a certain amount of pure water to make their bladders plentiful. Then they were placed in the supine position for conventional MR sequence scan and enhanced scan, including MRI plain scan first and then dynamic-enhanced scan. Philips 1.5T MR Imager was used, body coils were used as scanning transmitting coil. The scan started from the upper edge of the ilium to the bilateral femoral neck. A plain MRI scan for the first time, and then a dynamic enhanced scan for the second time (axial surface was FET1WI sequence, TR was 550 ms, TE was 9 ms, NSA was 2 times, thickness of layer was 6 mm, interlayer spacing was 2 mm; axial and sagittal surfaces were T2WI sequence, TR was 4000 ms, TE was 85 ms, NSA was 4 times, thickness of layer was 6 mm, interlayer spacing was 2 mm; axial, sagittal, and crown surfaces were FSET1WI sequence, TR was 5,000 ms, TE was 75 ms, NSA was 2 times). The images were reviewed by a radiologist with 7 years of experience in pelvic MRI diagnosis and a senior radiologist with 20 years of pelvic MRI diagnosis, for the comprehensive assessment according to the characteristics of the size, shape, apparent dispersion coefficient (ADC), signal uniformity and edge burrs of lymph nodes.

### Examination of tumor markers

Venous blood collected from each patient (3 mL) on an empty stomach within 2 weeks before surgery was centrifugated for serum, and then serum CA125 level was determined by Roche E70 full-automatic immunoanalyzer and chemiluminescence method, and serum SCCA level by Abbott i2000SR full-automatic immunoanalyzer and microparticle enzyme-linked immunoassay (MEIA), which were performed referring to the reagent instructions. The detection instruments were full-automatic chemiluminescence immunoanalyzers and supporting reagents, which were used strictly by the relevant operating criteria.

### Evaluation criteria

#### Staging criteria

The criteria for clinical staging were strictly in accordance with the FIGO whether the para-uterine, bladder, vagina, and so on have been violated. For MRI staging criteria, if there were soft tissue shadow, uneven or irregular cervical edge, but undamaged pelvic wall, it may be para-uterine infiltration, or it may be LNM when the short-axis diameter of T1 or T2 exceeded 10 mm. Comparing MRI scan with surgical pathology, the significance of MRI in para-uterine infiltration and LNM in cervical cancer were analyzed with sensitivity, specificity, and accuracy.

#### Positive criteria for tumor markers

Referring to relevant clinical criteria, reference critical value was normally 1.5 ng/L for SCCA, and 35.0 IU/mL for CA125. That beyond the normal reference critical value was considered as positive.

Positive lymph node criteria: short-axis diameter of lymph node > 10 mm.

#### Image analysis

Two senior imaging physicians were selected to review MRI images of all patients for retrospective review to observe the lesion, size, shape, infiltration, and ROC curve. ARSI (%) and MRSI (%) were calculated.

### Statistical methods

SPSS 19.0 software was used to process the data. The count data was expressed using the number of cases and percentage (%). Chi-square (*χ*^2^) test was used for single-factor comparison between groups. Measurement data was represented by (`*x*±*s*). Independent samples *t* test was used for single-factor comparison between groups. *α*=0.05 was used as the test level if there are no special instructions. *P*<0.05 indicated that the results were statistically significant. With postoperative pathological diagnosis as the gold criteria, the sensitivity, specificity, positive (negative) prediction rate, and accuracy were calculated and compared. Taking the sensitivity as the vertical axis and the specificity as lateral axis, ROC curve was drawn to evaluate the prediction value of tumor markers, then the area under the curve (expressed by AUC) was calculated, 0.5 < AUC ≤ 0.7 showed low diagnostic value, 0.7 < AUC ≤ 0.9 showed moderate diagnostic value, AUC > 0.9 showed high diagnostic value.

## Results

### Characteristics of patients

A total of 200 patients with cervical cancer were included in this study. Detailed patient baseline characteristics are shown in Table 1. Patients mostly aged 35–60 years (159 cases, 79.5%). The average age was 49.37±10.24 years old.

**Table 1:** Patient baseline characteristics

Characteristics		Ratio of patients
Age	< 35 years old	13 (6.5%)
	35–60 years old	159 (79.5%)
	> 60 years old	28 (14.0%)
Degree of tissue differentiation	High differentiation	6 (3.0%)
	Medium differentiation	49 (24.5%)
	Low differentiation	83 (41.5%)
	High-medium differentiation	11 (5.5%)
	Medium-low differentiation	51 (25.5%)
Diameter of tumors	> 4 cm	26 (13.0%)
	≤ 4 cm	174 (87.0%)
Depth of infiltration	> 1/2 muscular layer	138 (69.0%)
	≤ 1/2 muscular layer	62 (31.0%)
	Pulse canal infiltration	53 (26.5%)
	Vaginal involvement	17 (8.5%)

### MRI imaging performance in patients with cervical cancer

**Fig. 2:**
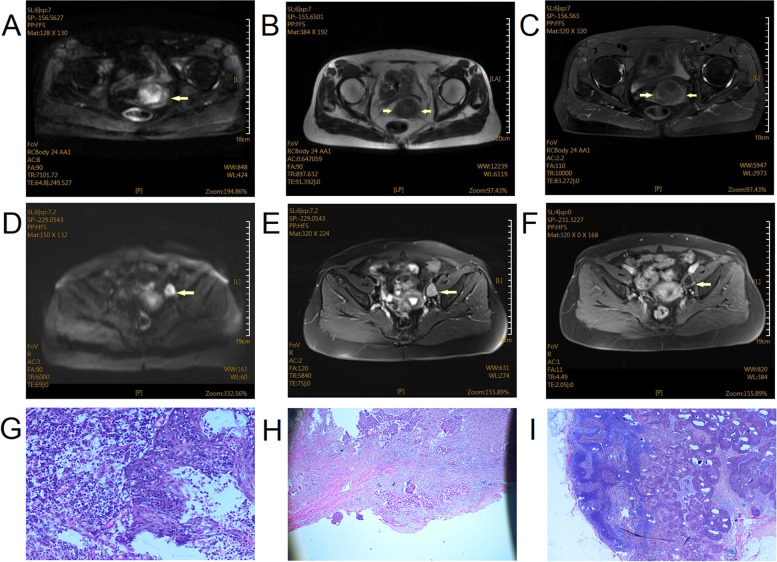
MRI imaging and pathological findings in patients with cervical cancer. **A** para-uterine infiltration DWI, **B** para-uterine infiltration T2WI, **C** para-uterine infiltration T2FS, **D** lymph node metastasis DWI, **E** lymph node metastasis T2FS, **F** lymph node metastasis enhancement, **G** cervical cancer pathology examination (uterine + double lateral attachment), **H** para-uterine infiltration pathology examination (uterus + double attachment), **I** lymph node metastasis pathology examination (uterus, bilateral fallopian tubes and left ovary)

Of the 200 patients with cervical cancer, MRI images of 14 patients with IB stage showing no positive characteristics (Fig. 2). Solid lumps were found in the other 186 patients with cervical cancer, with the diameter of lump within 0.60~7.82 cm. Moreover, there were 51 patients with the diameter of lesion > 4cm, 135 patients with the diameter of lesion ≤ 4cm, 169 patients with irregular lump, and 17 patients with regular lump, respectively. In addition, there were also 94 patients with para-uterine infiltration, and 37 patients with LNM. As shown in Fig. 2A–C, the structure of the lymph node hilum is visible, indicating that it is a benign lymph node without lymph node metastasis. Figure 2D–F, MRI images show that the diameter of the lymph node is greater than 10 mm. After the enhancement, the ring strengthens, the lymph node hilum disappears, the lymph node shape is round, the bound of the cortex and the medulla are not clear, the lymph node, the cortex is eccentrically thickened (≥0.3 cm), the structure of the lymphatic hilum disappears, and the cortex of the lymph node is located under the envelope of the lymph node, indicating that there is lymph node metastasis. The sensitivity, specificity, accuracy, positive prediction rates, and negative prediction rates of MRI diagnosis were 45.7, 92.3, 86.1, 65.4, and 82.9%, respectively, compared with the pathological results.

### Comparison of MRI staging, clinical staging, and postoperative pathological results

Of the 200 patients with cervical cancer, there were 165 patients with stage IB (82.5%), 26 patients with stage IIA (13.0%), 7 patients with stage IIB (3.5%), and 2 patients with stage IIIB (1%). There were 169 cases consistent with pathological results: 148 patients with stage IB, 17 patients with stage IIA, 4 patients with stage IIB, and 0 patients with stage IIIB according to MRI results.

### MRI diagnosis of lymph node metastasis

Of the 200 patients, there were 29 patients with LNM confirmed by pathological results, and according to the accurate diagnosis of MRI, 16 patients showed positive, and 15 false positive (MRI images showed that the diameter of lymph node was greater than 10 mm, with ring enhancement. Surgical examination found that although the lymph nodes were enlarged but soft, with no adhesion to the blood vessels, and postoperative pathology confirmed the swollen lymph nodes as an inflammatory reaction or hyperplasia), 6 false negative, and 163 negative. The sensitivity, specificity, accuracy, positive predictive rates, and negative MRI diagnostic rates were 55.2% (16/29), 91.6% (163/178), 89.5% (179/200), 51.6% (16/31), 96.4% (163/169), overrated 7.5% (15/200), and underrated 3% (6/200).

Morphological evaluation of MRI images showed that a significant number of patients were misdiagnosed, especially with low sensitivity (55.2%), which may be false negative due to small LNM. The result is consistent with several previous studies. Although pelvic anterior lymph node biopsy provided an effective means for the diagnosis of LNM, it is still invasive, which is limited to the detection of minor LNM.

### Relationship between SCCA and CA125 levels and lymph node metastasis

A total of 124 subjects were tested for serum SCCA level, including 19 with positive nodes, and 105 negative, with significant differences in SCCA levels between the two groups (*P* <0.01).

Moreover, 107 subjects were tested for serum CA125 level, 23 of which had positive nodes, 84 negative, and there was no significant difference in CA125 level between the two groups (*P* > 0.01), as shown in Table 2.

**Table 2:** SCCA and CA125 levels in patients with positive and negative lymph node metastasis

Group	Number of patients	SCCA (ng/mL)				Number of patients	CA125 (U/mL)			
		P25	P50	P75	*P*		P25	P50	P75	*P*
Positive	19	1.14	3.83	7.64	0.009	23	30.04	43.73	55.78	0.233
Negative	105	0.48	1.03	2.16		84	17.93	24.98	35.96	
Merge	124					107				

**Fig. 3:**
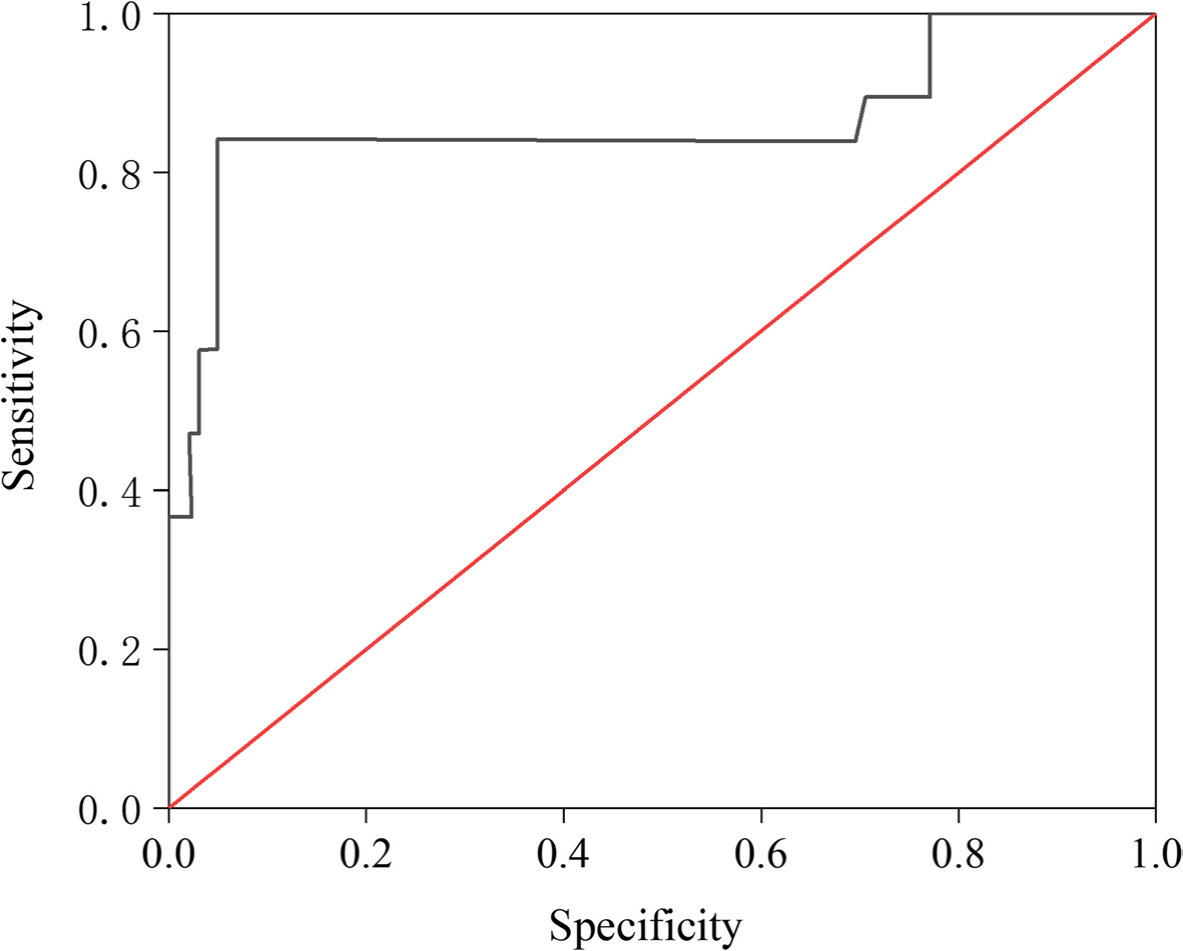
ROC curve of serum SCCA level for diagnosis of lymph node metastasis

The ROC curve (Fig. 3) can be used for evaluating the diagnosis of LNM. When the maximum critical value of the sum of sensitivity and specificity for serum SCCA was 3.46 ng/mL, the sensitivity to diagnose LNM was 58.1% and specificity 84.2%, the area under the ROC curve was 0.864. The standard error was 0.062, and the 95% confidence interval was 0.743–0.986, indicating that serum SCCA has a good diagnostic value for predicting LNM.

### Relationship between serum SCCA and CA125 levels and para-uterine infiltration

A total of 124 subjects were tested for serum SCCA, including 38 with positive para-uterine infiltration, and 86 negative, with significant differences in serum SCCA levels between the two groups (*P* <0.05).

Moreover, 107 patients were tested for serum CA125 level, including 43 with positive para-uterine infiltration and 64 negative, with no significant difference in serum CA125 level between the two groups (*P* > 0.05), as shown in Table 3.

**Table 3:** Serum SCCA and CA125 levels in patients with positive and negative para-uterine infiltration

Group	Number of patients	SCCA (ng/mL)				Number of patients	CA125 (U/mL)			
		P25	P50	P75	*P*		P25	P50	P75	*P*
Positive	38	4.14	7.65	9.98	0.041	43	10.17	14.28	35.34	0.873
Negative	86	0.57	1.23	3.78		64	9.03	13.12	19.14	
Merge	124					107				

**Fig. 4:**
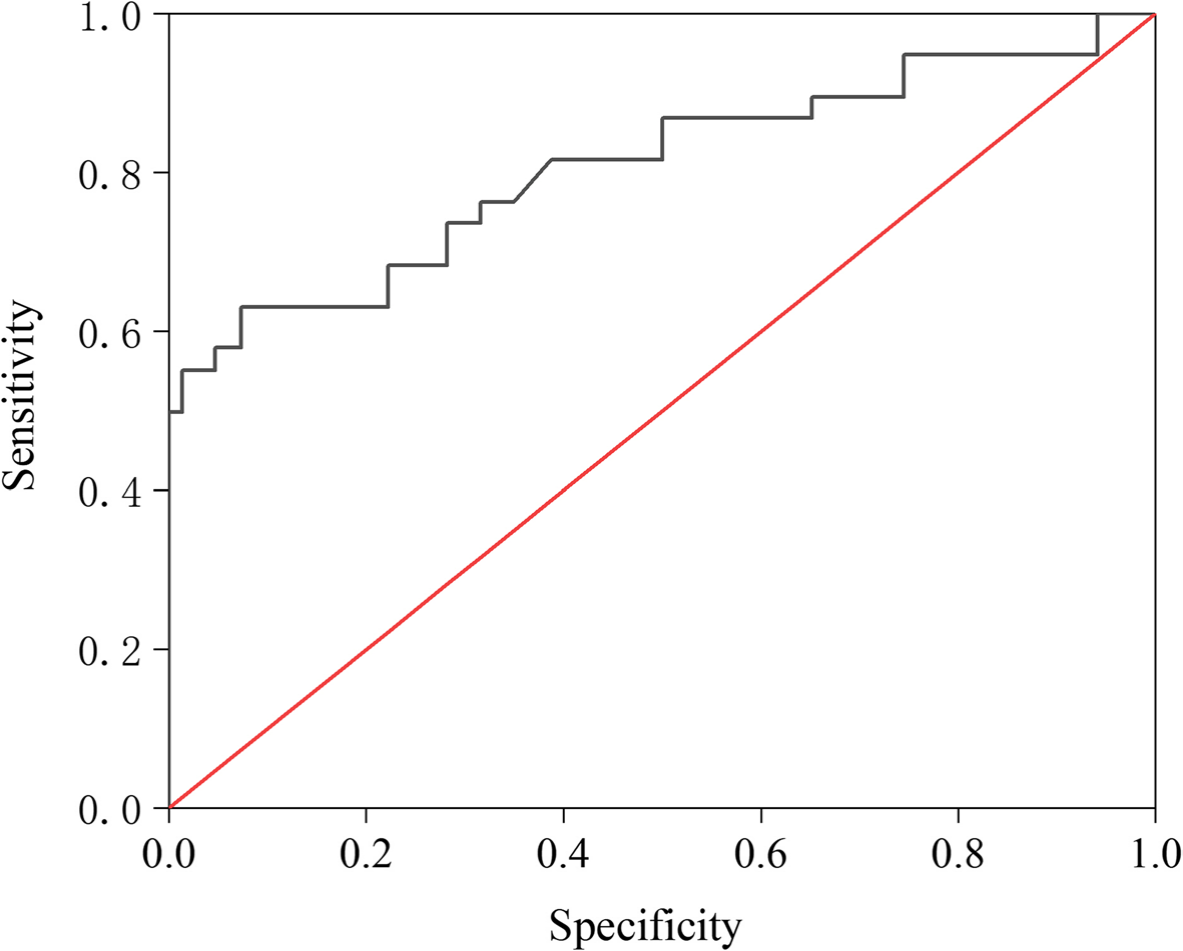
ROC curve of serum SCCA

The ROC curve (Fig. 4) was used to evaluate the clinical value of the diagnosis for serum SCCA level. When the maximum critical value of the sum of sensitivity and specificity for serum SCCA was 4.4 ng/mL, the sensitivity was 54.7%, specificity was 86.8%, and AUC of the ROC curve was 0.808, indicating a higher diagnostic value of serum SCCA for para-uterine infiltration (AUC > 0.7).

### Comparison of predicted positive rate in the diagnosis of cervical cancer between MRI and MRI combined with serum SCCA

The positive rate of tumor marker CA125 in the diagnosis for cervical cancer was slightly higher than that of SCCA and CA152, with no statistically significant difference (*P* > 0.05), that of MRI diagnosis for cervical cancer was 96.15%, which was higher than all tumor markers (*P* < 0.05). The accuracy of tumor markers combined with MRI in the diagnosis for cervical cancer was higher than that of MRI, with no statistically significant difference (*P* > 0.05), as shown in Table 4.

**Table 4:** Comparison of MRI and MRI combined with serum SCCA in 2018 patient-based FIGO staging

Characteristics	Indexes	MRI	MRI and SCCA
Primary tumor	Sensitivity	78.5% (157/200)	86.5% (173/200)
	Accuracy	56.0% (112/200)	71.5% (143/200)
IB	Sensitivity	98.8% (163/165)	100% (165/165)
	Accuracy	88.6% (31/35)	94.2% (33/35)
	Specificity	97.0% (194/200)	99.0% (158/200)
IIA	Sensitivity	100% (26/26)	100% (26/26)
	Accuracy	94.8% (165/174)	94.8% (165/174)
	Specificity	95.5% (191/200)	95.5% (25/200)
IIB	Sensitivity	85.7% (6/7)	100% (7/7)
	Accuracy	92.7% (179/193)	96.9% (187/193)
	Specificity	92.5% (185/200)	97.0% (194/200)
IIIB	Sensitivity	100% (2/2)	100% (2/2)
	Accuracy	91.4% (181/198)	97.0% (192/198)
	Specificity	91.5% (183/200)	97.0% (194/200)

### Diagnosis of lymph node metastasis and para-uterine infiltration in MRI combined with serum SCCA

With the serum SCCA bound value as 3.46 ng/mL, Lymph node metastasis in 124 patients underwent MRI examination and serum SCCA test as follows. Of the patients with positive lymph nodes, 12 were positive in the combined detection, and 7 negative in single detection. Moreover, of those with negative lymph nodes, 101 of them were negative in the combined detection, and 4 positive in single detection. The sensitivity and specificity of MRI combined with serum SCCA in the diagnosis for LNM were better than that of single auxiliary examination (Table 5).

**Table 5:** Diagnosis of lymph node metastasis in MRI, serum SCCA, MRI combined with serum SCCA

	Sensitivity	Specificity	Accuracy	Positive prediction rate	Negative prediction rate
MRI	55.2%	91.6%	89.5%	51.6%	96.4%
Serum SCCA	58.1%	84.2%	93.9%	53.1%	95.5%
Union	63.2%	96.0%	95.1%	56.4%	98.2%

With the serum SCCA bound value as 4.4 ng/mL, para-uterine infiltration in 124 patients underwent MRI examination and serum SCCA test as follows. Of patients with positive para-uterine infiltration, 29 were positive when the two were combined for use, and 9 negative in one of the two. Moreover, of patients with negative para-uterine infiltration, 83 were positive in the combined detection, and 3 positive in single detection. The sensitivity and specificity of MRI combined with serum SCCA in the diagnosis of peri-uterine infiltration were better than that of single auxiliary examination (Table 6).

**Table 6:** Diagnosis of para-uterine infiltration in MRI, serum SCCA, MRI combined with serum SCCA

	Sensitivity	Specificity	Accuracy	Positive prediction rate	Negative prediction rate
MRI	56.9%	74.1%	90.7%	43.5%	92.2%
Serum SCCA	54.7%	86.8%	92.7%	46.1%	91.9%
Merge	76.3%	95.3%	94.3%	54.7%	97.5%

## Discussion

The incidence of cervical cancer ranks second among female malignant tumors. There are nearly 400,000 cervical cancer patients in China, and the prevalence and fatality rates have increased significantly in recent years. The fatality rate is 11.3%, which is much higher than the 5% in developed countries [26]. Lymph node metastasis is a common dissemination form of cervical cancer. Its risk factors and metastasis are very important for formulating treatment plans and evaluating prognosis.

The methods currently used for clinical diagnosis of cervical cancer include colposcopy, cervical smear, liquid-based cytology and imaging screening methods, etc. Cervical smear and liquid-based cytology are all invasive examinations. Under some conditions, cervical biopsy is difficult to determine the status of tumor invasion. MRI is the preferred imaging method for cervical cancer screening. Its soft tissue resolution is high. Compared with abdominal and chest screening, the patient’s pelvic tissue is less affected by intestinal peristalsis, breathing, and other artifacts, and the pelvic pathological structure is clearly displayed. Early diagnosis of cervical cancer has good guiding value. MRI can realize multi-sequence and multi-directional imaging, which can provide a basis for cervical cancer staging and treatment, and it can lay an imaging foundation for accurately determining pelvic organ invasion and lymph node metastasis [27].

The sensitivity and specificity of MRI in the diagnosis for the depth of muscular invasion and the degree of cervical invasion in endometrial carcinoma can reach more than 80%. With the continuous improvement of MRI technology, multiplex mode MRI is gradually applied to the diagnosis of endometrial cancer, showing high application efficiency in clarifying myoscular infiltration depth, infiltration of cervical cervix, and preoperative staging [28].

Through the study of MRI scanning technology, we found that MRI scanning technology has a good effect on the prediction of cervical cancer invasion depth and lymphatic metastasis. Taking the postoperative pathological results as the gold standard, comparing the preoperative MRI scan results of cervical cancer with the clinical pathological results, the overall matching rate reached 82.5%. In addition, in terms of predicting para-uterine invasion of cervical cancer and predicting lymph node metastasis, the sensitivity, specificity, and accuracy of MRI scans exceed the preoperative clinical pathological staging. Therefore, MRI scans are useful for preoperative diagnosis of infiltration and lymph node metastasis in cervical cancer.

Tumor markers are effective means for early prediction of cancer occurrence, treatment, and prognosis. Among them, SCCA and CA125 are commonly used serum tumor markers for cervical cancer [29]. Among them, SCCA is mainly present in the cancer cell antigen in cervical squamous cell carcinoma, but SCCA expression is rare in normal human squamous epithelial tissue. SCCA is a highly specific serum tumor marker for squamous cell carcinoma, which means that SCCA has a better predictive value for tumors originating from squamous cells, such as esophageal cancer and cervical cancer, and the higher the clinical stage, the higher the SCCA increases. CA125 protein is an important antigen for ovarian cancer, and it is mostly used in the early prognosis of ovarian cancer and cervical cancer [30–32]. This study found that the SCCA level of patients with para-uterine invasion and lymph node metastasis of cervical cancer was significantly higher than that of patients without para-uterine invasion and lymph node metastasis (*P*<0.05), while there was no significant difference in the level of CA125, which indicates the presence of para-uterine invasion. Cervical cancer patients with metastasis to pelvic lymph nodes can be expressed in serum tumor marker levels to a certain extent, and the convenience of early clinical detection is strong, which helps to screen and grasp the degree of tumor progression as soon as possible. But at present, clinical changes in tumor marker levels are mostly used as an auxiliary means of tumor malignancy and patient treatment and prognosis. Choi et al. [33] showed that the lymph node metastasis and the diameter of malignant lesions are significantly related to the increase in peripheral blood SCCA levels. A study revealed that [34] the use of tumor markers in MRI-assisted screening can increase the detection rate of cervical cancer. This study found that the use of MRI scanning technology combined with serum SCCA levels is superior to MRI scanning technology alone in determining para-uterine invasion and lymph node metastasis. Although MRI has the disadvantages of overestimating tumor size and local invasion, the results showed that MRI combined with serum SCCA has greater value in FIGO staging and diagnosis in 2018 than MRI alone. MRI examination has good specificity and can make up for the defect of low specificity of serum tumor marker detection to a certain extent. Serum tumor marker detection can provide clinical diagnosis and disease evaluation information from the pathophysiological level, and at the same time help to improve the detection of small lesions. The combination of the two can complement each other’s disadvantages.

## Conclusions

In summary, the serum SCCA and CA125 tumor markers of patients with para-uterine invasion and pelvic lymph node metastasis of cervical cancer are increased compared with those of cervical cancer patients without para-uterine invasion and pelvic lymph node metastasis. Among them, SCCA has significant differences, and its accuracy rate is lower than that of MRI screening. MRI can be used as the first choice for cervical cancer staging, but for patients with cervical cancer that cannot be diagnosed clearly by MRI, tumor marker examinations can be supplemented to improve the accuracy of cervical cancer diagnosis.

## Data Availability

The datasets used and/or analyzed during the current study are available from the corresponding author on reasonable request.

## References

[1] Siegel RL, Miller KD, Jemal A (2017). Cancer statistics, 2017. CA Cancer J Clin..

[2] Cuzick J, Arbyn M, Sankaranarayanan R, Tsu V, Ronco G, Mayrand MH, Dillner J, Meijer CJ (2008). Overview of human papillomavirus-based and other novel options for cervical cancer screening in developed and developing countries. Vaccine..

[3] Chen W, Zheng R, Baade PD, Zhang S, Zeng H, Bray F, Jemal A, Yu XQ, He J (2016). Cancer statistics in China, 2015. CA Cancer J Clin..

[4] Torre LA, Bray F, Siegel RL, Ferlay J, Lortet-Tieulent J, Jemal A (2015). Global cancer statistics, 2012. CA Cancer J Clin..

[5] Tsuyoshi H, Tsujikawa T, Yamada S, Okazawa H, Yoshida Y (2021). Diagnostic value of 18F-FDG PET/MRI for revised 2018 FIGO staging in patients with cervical cancer. Diagnostics..

[6] Bermudez A, Bhatla N, Leung E (2015). Cancer of the cervix uteri. Int J Gynaecol Obstet..

[7] Tsunoda AT, Marnitz S, Soares Nunes J, Mattos de Cunha Andrade CE, Scapulatempo Neto C, Blohmer JU, Herrmann J, Kerr LM, Martus P, Schneider A (2017). Incidence of histologically proven pelvic and para-aortic lymph node metastases and rate of upstaging in patients with locally advanced cervical cancer: results of a prospective randomized trial. Oncology..

[8] Ruengkhachorn I, Therasakvichya S, Warnnissorn M, Leelaphatanadit C, Sangkarat S, Srisombat J (2015). Pathologic risk factors and oncologic outcomes in early-stage cervical cancer patients treated by radical hysterectomy and pelvic lymphadenectomy at a Thai University Hospital: a 7 year retrospective review. Asian Pac J Cancer Prev..

[9] Ferrandina G, Pedone Anchora L, Gallotta V, Fagotti A, Vizza E, Chiantera V, De Iaco P, Ercoli A, Corrado G, Bottoni C (2017). Can we define the risk of lymph node metastasis in early-stage cervical cancer patients? A Large-Scale, Retrospective Study. Ann Surg Oncol..

[10] Achouri A, Huchon C, Bats AS, Bensaid C, Nos C, Lécuru F (2013). Complications of lymphadenectomy for gynecologic cancer. Eur J Surg Oncol..

[11] Jeong SY, Park H, Kim MS, Kang JH, Paik ES, Lee YY, Kim TJ, Lee JW, Kim BG, Bae DS (2020). Pretreatment lymph node metastasis as a prognostic significance in cervical cancer: comparison between disease status. Cancer Res Treat..

[12] Colombo N, Preti E, Landoni F, Carinelli S, Colombo A, Marini C, Sessa C, ESMO Guidelines Working Group (2011). Endometrial cancer: ESMO Clinical Practice Guidelines for diagnosis, treatment and follow-up. Ann Oncol..

[13] Yildirim N, Saatli B, Kose S, Sancar C, Ulukus C, Koyuncuoglu M, Saygili U, Obuz F (2018). Predictability of myometrial, lower uterine segment and cervical invasion with 3D transvaginal ultrasonography and magnetic resonance imaging in endometrial cancer patients: a prospective cohort study. Med Ultrason..

[14] Luo L, Luo Q, Tang L (2020). Diagnostic value and clinical significance of MRI and CT in detecting lymph node metastasis of early cervical cancer. Oncol Lett..

[15] Haldorsen IS, Salvesen HB (2012). Staging of endometrial carcinomas with MRI using traditional and novel MRI techniques. Clin Radiol..

[16] Decazes P, Hinault P, Veresezan O, Thureau S, Gouel P, Vera P (2021). Trimodality PET/CT/MRI and radiotherapy: a mini-review. Front Oncol..

[17] Hou L, Zhou W, Ren J, Du X, Xin L, Zhao X, Cui Y, Zhang R (2020). Radiomics analysis of multiparametric MRI for the preoperative prediction of lymph node metastasis in cervical cancer. Front Oncol..

[18] Song J, Hu Q, Ma Z, Zhao M, Chen T, Shi H (2021). Feasibility of T2WI-MRI-based radiomics nomogram for predicting normal-sized pelvic lymph node metastasis in cervical cancer patients. Eur Radiol..

[19] Balcacer P, Shergill A, Litkouhi B (2019). MRI of cervical cancer with a surgical perspective: staging, prognostic implications and pitfalls. Abdom Radiol (NY)..

[20] Choi HJ, Ju W, Myung SK, Kim Y (2010). Diagnostic performance of computer tomography, magnetic resonance imaging, and positron emission tomography or positron emission tomography/computer tomography for detection of metastatic lymph nodes in patients with cervical cancer: meta-analysis. Cancer Sci..

[21] Liu SC, Huang EY, Hu CF, Ou YC, ChangChien CC, Wang CJ, Tsai CC, Fu HC, Wu CH, Lin H (2016). Pretreatment factors associated with recurrence for patients with cervical cancer international federation of gynecology and obstetrics stage IB1 disease. Gynecol Obstet Invest..

[22] Guo S, Yang B, Liu H, Li Y, Li S, Ma L, Liu J, Guo W (2017). Serum expression level of squamous cell carcinoma antigen, highly sensitive C-reactive protein, and CA-125 as potential biomarkers for recurrence of cervical cancer. J Cancer Res Ther..

[23] Zamani N, Modares Gilani M, Zamani F, Zamani MH (2015). Utility of pelvic MRI and tumor markers HE4 and CA125 to predict depth of myometrial invasion and cervical involvement in endometrial cancer. J Family Reprod Health..

[24] Attia EF, Jolley SE, Crothers K, Schnapp LM, Liles WC (2016). Soluble vascular cell adhesion molecule-1 (sVCAM-1) is elevated in bronchoalveolar lavage fluid of patients with acute respiratory distress syndrome. PLoS One..

[25] Armangue T, Leypoldt F, Málaga I, Raspall-Chaure M, Marti I, Nichter C, Pugh J, Vicente-Rasoamalala M, Lafuente-Hidalgo M, Macaya A (2014). Herpes simplex virus encephalitis is a trigger of brain autoimmunity. Ann Neurol..

[26] McGlynn KA, Petrick JL, London WT (2015). Global epidemiology of hepatocellular carcinoma: an emphasis on demographic and regional variability. Clin Liver Dis..

[27] Théodore C, Levaillant JM, Capmas P, Chabi N, Skalli D, Vienet-Legué L, Haddad B, Fernandez H, Touboul C (2017). MRI and ultrasound fusion imaging for cervical cancer. Anticancer Res..

[28] Tait LM, Hoffman D, Benedict S, Valicenti R, Mayadev JS (2016). The use of MRI deformable image registration for CT-based brachytherapy in locally advanced cervical cancer. Brachytherapy..

[29] Kontostathi G, Zoidakis J, Anagnou NP, Pappa KI, Vlahou A, Makridakis M (2016). Proteomics approaches in cervical cancer: focus on the discovery of biomarkers for diagnosis and drug treatment monitoring. Expert Rev Proteomics..

[30] Sturgeon CM, Duffy MJ, Hofmann BR, Lamerz R, Fritsche HA, Gaarenstroom K, Bonfrer J, Ecke TH, Grossman HB, Hayes P (2010). National Academy of Clinical Biochemistry Laboratory Medicine Practice Guidelines for use of tumor markers in liver, bladder, cervical, and gastric cancers. Clin Chem..

[31] Sasaki A, Akita K, Ito F, Mori T, Kitawaki J, Nakada H (2015). Difference in mesothelin-binding ability of serum CA125 between patients with endometriosis and epithelial ovarian cancer. Int J Cancer..

[32] Boichenko AP, Govorukhina N, Klip HG, van der Zee AG, Güzel C, Luider TM, Bischoff R (2014). A panel of regulated proteins in serum from patients with cervical intraepithelial neoplasia and cervical cancer. J Proteome Res..

[33] Choi KH, Lee SW, Yu M, Jeong S, Lee JW, Lee JH (2019). Significance of elevated SCC-Ag level on tumor recurrence and patient survival in patients with squamous-cell carcinoma of uterine cervix following definitive chemoradiotherapy: a multi-institutional analysis. J Gynecol Oncol..

[34] Zou H, Zhou Y (2021). Diagnostic value of CT combined with high resolution pelvic MRI in colorectal cancer. Imaging Science and Photochemistry..

